# Construction of Z-Scheme ZIF67/NiMoO_4_ Heterojunction for Enhanced Photocatalytic Degradation of Antibiotic Pollutants

**DOI:** 10.3390/ma17246225

**Published:** 2024-12-20

**Authors:** Kandasamy Sasikumar, Ramar Rajamanikandan, Heongkyu Ju

**Affiliations:** Department of Physics, Gachon University, Seongnam-si 13120, Gyeonggi-do, Republic of Korea; sasiphy2022@gachon.ac.kr (K.S.); chemistrmkd@gachon.ac.kr (R.R.)

**Keywords:** antibiotic pollutants, NiMoO_4_, photocatalysis, visible light, ZIF67

## Abstract

The rational design of heterojunction photocatalysts enabling fast transportation and efficient separation of photoexcited charge carriers is the key element in visible light-driven photocatalyst systems. Herein, we develop a unique Z-scheme heterojunction consisting of NiMoO_4_ microflowers (NMOF) and ZIF67, referred to as ZINM (composite), for the purpose of antibiotic degradation. ZIF67 was produced by a solution process, whereas NMOF was synthesized via coprecipitation with a glycine surfactant. The NMOF exhibited a monoclinic phase with a highly oriented, interconnected sheet-like morphology. The ZINM showed better optical and charge transfer characteristics than its constituents, ZIF67 and NiMoO_4_. Consequently, the developed heterojunction photocatalysts exhibited superior photocatalytic redox capability; the ZINM30 (the composite with 30 wt.% of NiMoO_4_ loaded) could degrade 91.67% of tetracycline and 86.23% of norfloxacin within 120 min. This enhanced photocatalytic activity was attributable to the reduced bandgap (E_gap_ = 2.01 eV), unique morphology, high specific surface area (1099.89 m^2^/g), and intimate contact between ZIF67 and NiMoO_4_, which facilitated the establishment of the Z-scheme heterojunction. Active species trapping tests verified that •O_2_^−^ and h^+^ were the primary species, supporting the proposed degradation mechanism. This work highlights a valid Z-scheme ZIF67/NiMoO_4_ heterojunction system for efficient carrier separation and, therefore, enhanced photocatalytic degradation of antibiotics.

## 1. Introduction

Clean water is one of the essential elements for the sustainability and preservation of ecological systems on Earth. In recent years, water pollution has become a serious threat to both humans and the environment [1,2,3]. One of the prime concerns for water pollution is the excessive and improper release of antibiotics by the pharmaceutical industries, hospitals, and agriculture sectors [4]. Tetracycline (TCE), a highly consumed antibiotic, is commonly used in human disease treatment, aquaculture, and animal husbandry [5]. Norfloxacin (NF) is a third-generation quinolone antibiotic extensively used in anti-inflammatory and antibacterial therapies [6]. These antibiotics are difficult to degrade by microorganisms in nature. As a result, the accumulation of their residues in soil and water can cause antibiotic resistance in bacteria, endangering human and environmental health [7]. This, in turn, has impelled researchers to adopt effective treatment technology to eliminate the abovementioned antibiotic pollutants in water bodies.

Semiconductor (SC) photocatalytic systems have garnered attention due to their remarkable catalytic efficacy, robust oxidation capacity, superior chemical stability, environmental friendliness, and mild reaction conditions [8]. Single-component photocatalysts suffer from either inefficient absorption of visible light or a high rate of recombination of photoexcited electron–hole (e^−^-h^+^) pairs. Instead, in the conventional type-II heterojunction photocatalysts, the conduction band (CB) position of SC-1 is more negative than that of SC-2, and the valence band (VB) position of SC-2 is more positive than SC-1. Under light exposure, the photoinduced electrons in the CB of SC-I transfer to the CB of SC-II, and the photoexcited holes in the VB of SC-II move to the VB of SC-I. As a result, the photogenerated electrons in the lower CB position of SC-2 could participate in the reduction process, while the photogenerated holes in the higher VB position of SC-1 could be involved in the oxidation process. This could lead to a weak redox capacity with electrons and holes [9]. On the other hand, in the direct Z-scheme heterojunction, the electrons in the lower CB of SC-2 recombine with the holes present in the higher VB position of SC-1. This process would eliminate electrons of weak redox potential while retaining those of strong ones in the more negative CB of SC-1 and holes in the more positive VB of SC-2, retaining their strong reduction–oxidation potentials. Thus, direct Z-scheme heterojunction could lead to enhanced spatial separation of photoinduced charge carriers and high charge carrier lifespan while maintaining strong redox capacity [10,11].

Metal–organic frameworks (MOFs) are two- or three-dimensional porous crystalline structures where metal ions are interlinked by organic ligands [12,13]. ZIF67, one of the zeolitic imidazolate frameworks (ZIF) in the branch of MOFs, possesses a high specific surface area, distinct morphologies, good chemical stability, and excellent thermal stability (>550 °C in N_2_ atmosphere). Its relatively narrow band gap (E_gap_ ~ 1.98 eV) offers strong visible light absorption capability, while its high porosity affords abundant active sites for photocatalytic reactions. Additionally, under ambient conditions, ZIF67 can easily self-assemble into homogenous mesoporous structures at a large scale [14]. However, it has the drawbacks of poor conductivity and fast e^−^-h^+^ pair recombination. Therefore, ZIF67 is frequently combined with other semiconductors to boost its photocatalytic activity [15].

Over the past few decades, molybdate-based semiconductor photocatalysts, including ZnMoO_4_, NiMoO_4_, Bi_2_MoO_6_, and CeMo_2_O_8_, have been continually developed because of their exceptional photocatalytic activity [16,17,18,19]. NiMoO_4_ has a moderate band gap (~2.3–2.8 eV) and an appropriate energy band structure, utilizing visible light for CO_2_ conversion, H_2_ production, and environmental pollutant degradation [20,21,22,23]. Additionally, it is affordable and has environmentally friendly features. Despite the intriguing properties, the photocatalytic performance of NiMoO_4_ is restrained by the quick recombination of photoexcited charge carriers and poor specific surface area [20]. Therefore, developing the Z-scheme heterojunction of NiMoO_4_ may be a beneficial approach to boost its photocatalytic activity.

To date, only a few sets of Z-scheme heterojunctions have been designed by coupling NiMoO_4_ with other photocatalysts. Nguyen et al. reported direct Nb-NiMoO_4_/g-C_3_N_4_ Z-heterojunction with high stability for CO_2_ conversion [24]. We also previously reported the improved photocatalytic properties of Z-scheme g-C_3_N_4_/NiMoO_4_ heterojunction toward pharmaceutical (ciprofloxacin) and dye (malachite green) pollutants [25]. Nevertheless, to the best of our knowledge, the synthesis of a Z-scheme heterojunction with the combination of NiMoO_4_ and ZIF67 for photocatalytic degradation of environmental pollutants has not yet been reported. Such a combination is anticipated to have an appropriate energy band structure that prevents carrier recombination [26,27,28].

Motivated by the aforementioned viewpoints, we fabricated a Z-scheme ZIF67/NiMoO_4_ heterojunction photocatalyst for the rapid degradation of antibiotics in water. ZIF67 was prepared through a solution process, while NiMoO_4_ microflowers (NMOF) were independently synthesized using glycine as a surfactant by a facile coprecipitation method. The NMOF possessed highly oriented and interconnected sheet-like structures. The ZIF67 and NMOF components were coupled together ultrasonically to form the heterojunctions. The effect of NiMoO_4_ loading (10, 20, 30 wt.%) on the photodegradation of pollutants was investigated. The unique morphology of NMOF significantly influenced the composites’ surface area, optical properties, and charge transport properties. The optimized composite, i.e., ZINM30 (the composite with 30 wt.% of NiMoO_4_ loaded), exhibited the highest photocatalytic degradation for TCE and NF. The role of photoactive species in the degradation processes was analyzed through scavenger studies. Based on the experimental findings, a suitable degradation mechanism was finally proposed.

## 2. Materials and Methods

### 2.1. Chemicals

Cobalt (II) nitrate hexahydrate (Co(NO_3_)_2_·6H_2_O, 98%), nickel (II) nitrate hexahydrate (Ni(NO_3_)_2_.6H_2_O, ≥97%), sodium molybdate dihydrate (Na_2_MoO_4_.2H_2_O, ≥99.5%), 2-methylimidazole (C_4_H_6_N_2_, 99%), glycine, tetracycline, and norfloxacin were supplied by Sigma Aldrich, Saint Louis, MO, USA. As the chemicals were analytical grade (AR), purification was unnecessary. Ultrapure water (18–19 μS/cm conductivity) was received from Millipore, Burlington, MA, USA.

### 2.2. Preparation of ZIF67

ZIF67 was prepared as follows. Briefly, 3.492 g of cobalt nitrate hexahydrate and 3.936 g of 2-methylimidazole were dissolved separately in 50 mL of methanol (solutions 1 and 2). Following a gentle magnetic stirring, solution 1 was gradually added dropwise into solution 2 and stirred further for approximately 2 h at room temperature to obtain a homogeneous solution. The mixture solution was preserved at 25 °C in a dark condition. After 24 h, the mixture was centrifuged (8000 rpm) twice with methanol to remove the unreacted chemicals. The deposit was collected, dried overnight in a vacuum at 70 °C, and marked as ZIF67.

### 2.3. Preparation of NiMoO_4_ Microflowers

NiMoO_4_ microflowers (NMOF) were synthesized via the coprecipitation method. Nickel nitrate hexahydrate (50 mmol, 1.017 g) and sodium molybdate dihydrate (50 mmol, 0.846 g) were dissolved in 70 mL of water. Glycine (400 mmol, 2.101 g) was added to this mixture. The final turquoise blue color solution was gently stirred. Then, NaOH (10 M, 10 mL) was added dropwise into the above solution to reach the pH ~ 13. With continuous stirring, the solution was heated at 80 °C for 30 min and kept without disturbance. After a few hours, a green precipitate was produced and collected by centrifugation. The precipitate was washed twice in water and anhydrous ethanol to reach a neutral pH. After vacuum-drying at 60 °C, the product was calcined at 500 °C (ramp rate of 5 °C/min) for 3 h in a muffle furnace and labeled as NMOF.

### 2.4. Preparation of ZINM Heterojunctions

For the preparation of ZINM heterojunctions, the mass ratio of ZIF67 was constant, and that of NMOF was varied. Three dispersions of ZIF67 were made in 50 mL of ethanol. With this, different masses of NMOF (10, 20, and 30 wt.%) were mixed and ultrasonicated for 3 h to obtain homogeneous composites. Then, the composites were stirred well at 60 °C for several hours to remove ethanol and dried overnight. The products were ground in a mortar and finally marked as ZINM10, ZINM20, and ZINM30. The complete synthesis procedure for NMOF, ZIF67, and ZINM photocatalysts is displayed in Figure S1.

### 2.5. Characterization Methods

The characterization methods, photoelectrochemical measurements, and photocatalytic activity tests are presented in the Supporting Information File.

## 3. Results and Discussion

### 3.1. Crystal Structure

**Figure 1:**
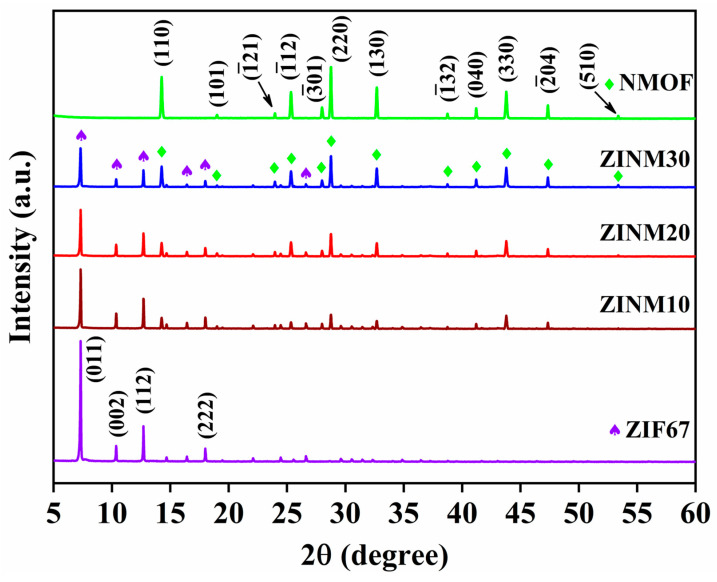
PXRD patterns of ZIF67, NMOF, ZINM10, ZINM20, and ZINM30 photocatalysts. The symbols “♠” and “♦” represent the diffraction peaks from ZIF67 and NMOF, respectively.

The PXRD data of ZIF67, NMOF, ZINM10, ZINM20, and ZINM30 are depicted in Figure 1. The typical and sharp diffraction peaks appearing at 7.4°, 10.4°, 12.8°, and 18.1° can be attributed to the (011), (002), (112), and (222) planes of ZIF67. The peaks were highly comparable to the previously reported data [29]. For NMOF, the characteristic peaks are found at 14.30°, 19.00°, 23.96°, 25.34°, 28.14°, 28.83°, 32.46°, 38.74°, 41.19°, 43.85°, 47.39°, and 53.38° corresponding to the (110), (101), (-121), (-112), (-301), (220), (130), (-132), (040), (330), (-204), and (510) lattice planes belongs to the monoclinic α-NiMoO_4_ with the space group of *I2/m* (12) (PDF card no.: 00-033-0948), respectively [30]. In the XRD pattern of ZINM composites, the peaks originating from ZIF67 and NMOF indicate that the composites were successfully formed. Compared to the peaks of individual ZIF67 and NMOF components, the peaks of ZIF67 gradually decrease with the increase in NMOF content in the composites. Meanwhile, the characteristic peaks of NMOF gradually increase. Moreover, peaks related to other residues or impurities are not observed, suggesting that the product is of high purity.

The structural properties of the as-prepared photocatalysts were evaluated by determining the crystalline parameters shown in Table S1. With the increase in NMOF loading (10, 20, and 30 wt.%) into the ZIF67 sample, the diffraction peaks become broader, as confirmed by the broadening in the FWHM values. This is indicative of the reduction in the crystallite size of the composites. The mean crystallite sizes were 14.28, 12.90, and 11.07 nm for ZINM10, ZINM20, and ZINM30, respectively. The reduced crystallite size of ZINM30 indicates its lower crystallinity. The small peak shift in the ZINM30 composite indicates the interaction of NiMoO_4_ with ZIF67 at the junction interface. The pure ZIF67 and NMOF photocatalysts exhibited microstrain values of 0.195 and 0.355 nm, respectively. The microstrain increased owing to the reduced crystallite size as the NMOF content increased in the composites, and the value reached its maximum (0.324 nm) for the ZINM30 composite (Table S1). The structural strain was induced due to the lattice mismatch in the composites, suggesting the heterostructure formation between ZIF67 and NMOF. The structural properties were also assessed from the Williamson–Hall (W-H) plots shown in Figure S2. The as-calculated D and ε values through the W-H method were slightly varied from the results of the Debye–Scherrer method, which could be due to the non-zero residual stress [31].

### 3.2. Functional Groups and Chemical States

**Figure 2:**
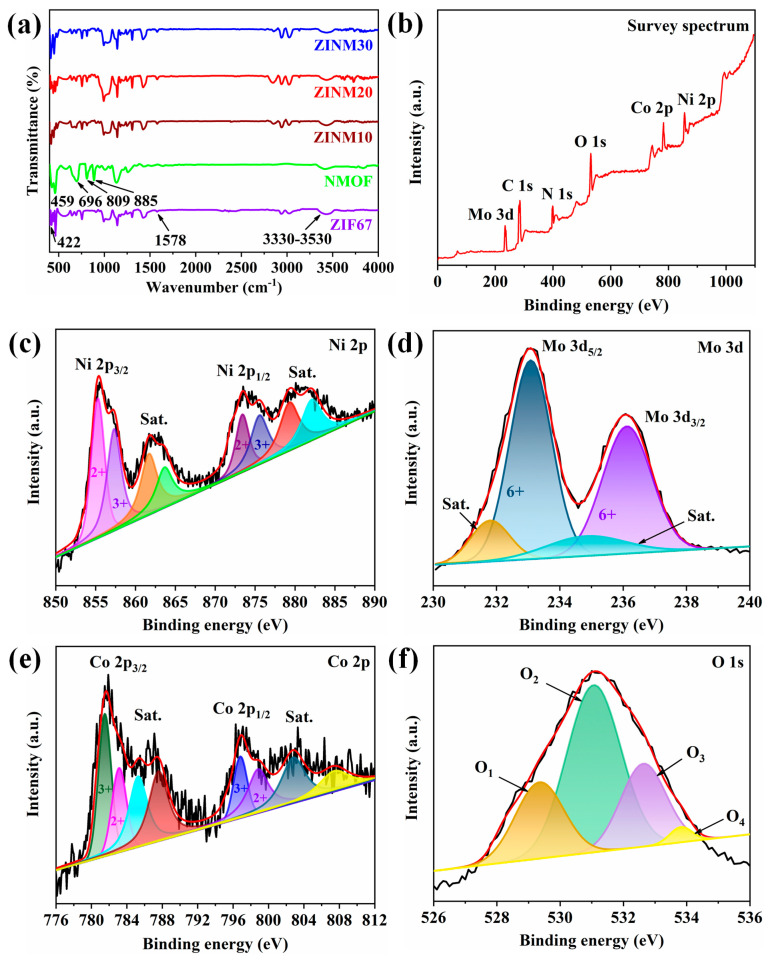
(**a**) FTIR spectra of the as-synthesized photocatalysts. (**b**) XPS survey spectrum. High-resolution spectra of (**c**) Ni 2p, (**d**) Mo 3d, (**e**) Co 2p, and (**f**) O 1s of ZINM30 photocatalyst.

FTIR spectra of the produced samples are displayed in Figure 2a. ZIF67 has a noticeable peak at 422 cm^−1^, corresponding to the Co–N moiety’s stretching vibration. The remaining peaks located in the 600–1500 cm^−1^ region are linked to the stretching and bending vibrations of the imidazole ring. The distinctive peak at 1578 cm^−1^ should be associated with the stretching vibration of C=N in the imidazole ring [32]. The broad band at 3330–3530 cm^−1^ is assigned to the O–H stretching vibration of surface-adsorbed H_2_O molecules. For NMOF, the characteristic peaks appearing below 1000 cm^−1^ are related to metal oxide (M–O) bonds. The band at 459 cm^−1^ could be produced by the Mo–O–Mo bond. The peaks at 696 and 809 cm^−1^ are perceptible and are associated with the Mo–O–Ni bond vibrations. The peaks at 885 and 948 cm^−1^ can be ascribed to the antisymmetric and symmetric stretching vibrations of the Mo=O bond [33]. The peak at 809 cm^–1^ in ZINM heterojunctions is substantially weaker than in NMOF, which could be due to the synergistic interaction between ZIF67 and NMOF. The distinctive peaks at 1304 cm^–1^ and 1424 cm^–1^ are greatly improved. This can be linked to the bending vibration of the pyrrole ring in ZIF67, which provides direct evidence for the effective recombination of the individual components [34].

Further studies on the chemical compositions and valence states of the heterojunctions were performed through XPS analysis. The survey spectrum of the ZINM30 (Figure 2b) consists of the prominent binding energy (BE) peaks of nickel (Ni 2p), molybdenum (Mo 3d), cobalt (Co 2p), oxygen (O 1s), carbon (C 1s), and nitrogen (N 1s) species. The high-resolution XPS spectrum of Ni 2p can be resolved into two spin-orbit components (ΔBE ∼ 18.1 eV), viz., Ni 2p_3/2_ and Ni 2p_1/2_ (Figure 2c). The peak maxima at binding energies of 855.3 and 873.4 eV are assigned to Ni 2p_3/2_ and Ni 2p_1/2_ of Ni^2+^ state, respectively. Similarly, the peaks at 857.3 and 875.6 eV are associated with 2p_3/2_ and 2p_1/2_ of Ni^3+^. The satellite peaks appear at 861.68, 863.63, 879.3, and 882.09 eV. The presence of Ni^3+^ could probably be due to unavoidable surface oxidation during the XPS sampling [35,36,37]. The high-resolution Mo 3d spectrum (Figure 2d) can be deconvoluted into two main peaks at 233.08 and 236.1 eV credited to Mo 3d_5/2_ and Mo 3d_3/2_, respectively. The binding energy difference of ΔBE ~ 3.02 eV implies the characteristic Mo^6+^ state. Two shake-up satellite peaks at 231.8 and 234.8 eV are associated with Mo^4+^ [38,39]. In the core-level spectrum of Co 2p (Figure 2e), two main peaks are seen at 781.51 and 796.81 eV with a spin-energy separation of 15.3 eV, corresponding to Co 2p_3/2_ and Co 2p_1/2_ of Co^3+^, respectively. The distinctive peaks at 783.13 and 798.82 eV are attributed to Co^2+^ [40,41]. In addition to that, the satellite peaks are observed at 785.28, 787.57, 802.79, and 807.41 eV related to Co^2+^. This confirms the coexistence of Co^3+^ and Co^2+^ in the ZINM30 photocatalyst. The deconvolution of the O 1s spectrum (Figure 2f) gives four peaks at BEs of 529.35, 531.06, 532.63, and 533.82 eV, which can be represented as O_1_, O_2_, O_3_, and O_4_, respectively. The O_1_ component arises due to the low-oxygen-coordinated defect sites (O^2−^) [42]. The O_2_ component is assigned to the typical metal–oxygen (M–O) bond [37,43]. The O_3_ and O_4_ components are contributed by the surface-adsorbed –OH/O_2_ groups and physic/chemisorbed H_2_O molecules on the material surface, respectively [44]. The high-resolution C 1s spectrum (Figure S2a) displays three distinct peaks, specifically at 284.5 eV, 286.2 eV, and 288.1 eV, indicating the existence of C–C sp^3^ hybridized carbon, C–OH, and O=C–O, respectively [40]. Similarly, the N 1s spectrum (Figure S2b) was deconvoluted into three peaks at 397.5, 399.1, and 400.7 eV, which corresponded to pyridinic-N, pyrrolic-N, and graphitic-N, respectively [41]. The above XPS results indicate that there is a strong interaction between NiMoO_4_ and ZIF67.

### 3.3. Morphological Properties

**Figure 3:**
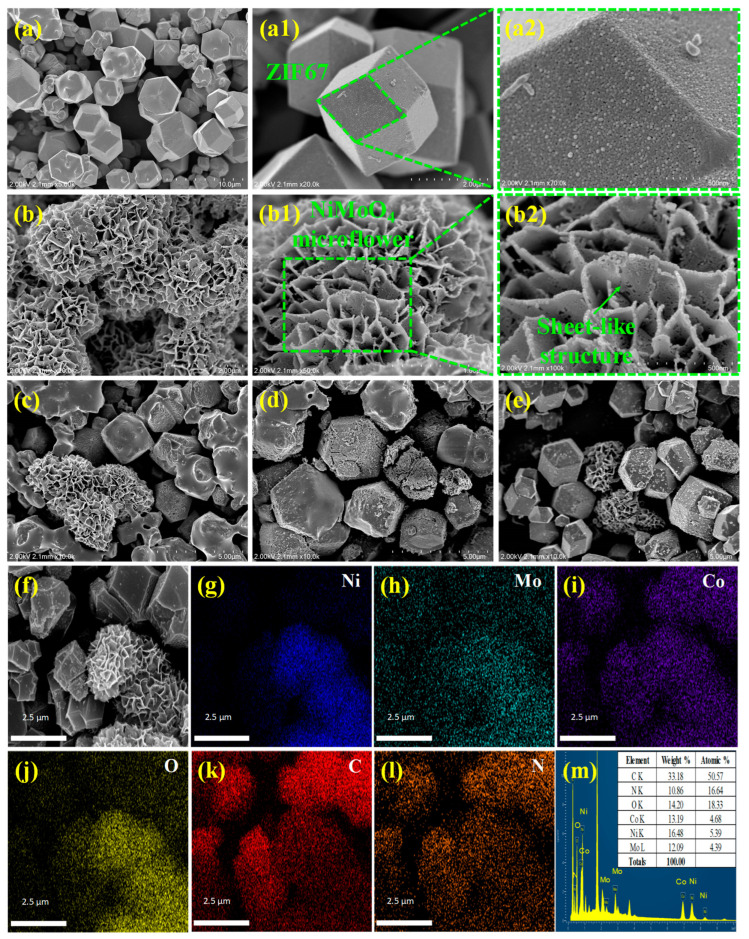
FESEM micrographs of (**a**–**a2**) ZIF67, (**b**–**b2**) NMOF, (**c**) ZINM10, (**d**) ZINM20, (**e**) ZINM30 photocatalysts. (**f**) FESEM image of ZINM30. (**g**–**l**) EDX mapping images corresponding to Ni, Mo, Co, O, C, and N elements in ZINM30. (**m**) EDX spectrum of ZINM30.

The morphology and microstructure of the photocatalysts were disclosed using a field-emission scanning electron microscope (FESEM), and the corresponding photographs are displayed in Figure 3. Figure 3a shows the FESEM picture of the as-synthesized ZIF67, which appears as a rhombic dodecahedral structure with a very smooth surface [45]. The particle size distribution histogram, depicted in Figure S3, indicates that the average particle size is about 3.5 μm. Figure 3(a1) shows the magnified image of a single ZIF67 particle. Figure 3(a2) illustrates the single facet of the particle, composed of several tiny spherical particles. The FESEM images of NMOF at different magnifications are displayed in Figure 3(b–b2). Figure 3b shows the densely packed hierarchical NiMoO_4_ microflowers. As seen in Figure 3(b1,b2), the microflowers are formed by interconnected, sheet-like structures with a thickness of around 21 nm. It appears that the NiMoO_4_ surface has voids, which might have resulted from the calcination process. Figure 3c–e shows ZIF67/NiMoO_4_ composites, whose surface becomes relatively rougher than that of pure ZIF67 as the NiMoO_4_ content increases from 10 to 30 wt.%, suggesting that NiMoO_4_ was successfully compounded with ZIF67. The EDX mapping images (Figure 3f–l) of ZINM30 inferred that the sample included Ni, Mo, Co, O, C, and N elements. The core elements of NMOF, including Ni, Mo, and O, were uniformly dispersed over the ZIF67 dodecahedron. This is further verified by the EDX spectrum, which is shown in Figure 3m. According to the above analysis, combining ZIF67 with NMOF would increase the transport of photoexcited electron–hole pairs and light absorption.

### 3.4. Specific Surface Area Measurements

**Figure 4:**
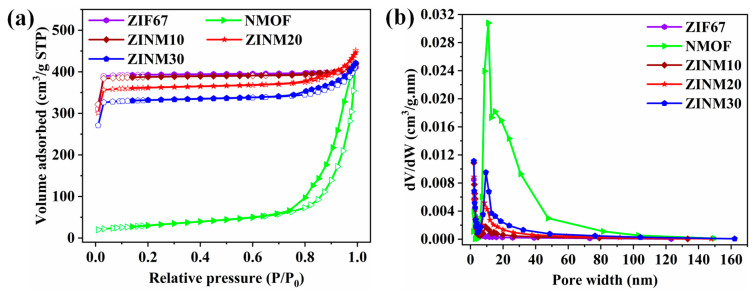
(**a**) N_2_ adsorption–desorption isotherms of ZIF67, NMOF, ZINM10, ZINM20, and ZINM30. (**b**) Pore size distribution curves of the photocatalysts.

The surface area and porous characteristics of photocatalyst materials were studied by measuring nitrogen (N_2_) adsorption–desorption isotherms, as depicted in Figure 4a. The corresponding pore size distribution curves are shown in Figure 4b. It can be seen in Figure 4a that the ZIF67 shows the type-1 isotherm, suggesting the 3D porous framework contains more micropores with a pore size of 2.03 nm (Figure 4b) [46]. The NMOF exhibit type-4 isotherm according to the IUPAC classification with an obvious hysteresis loop in the relative pressure range (P/P_0_) of 0.75–1.0, suggesting the presence of the mesoporous structure in the material (Figure 4b). Meanwhile, a combination of type-1 and type-4 hysteresis loops were observed in the isotherms of ZINM samples, prominently signifying the existence of both micro- and mesopores with monolayer–multilayer adsorption. The pore size distribution curves show the maximum peak in the narrow range of 8–10 nm corresponding to mesoporous structure. This improved mesoporous nature of the ZINM heterojunctions was due to the increase in NMOF content into ZIF67. The calculated BET surface area (S_BET_), pore width (D_Avg_), and pore volume (V_Total_) are listed in Table S2. The S_BET_ of ZIF67, NMOF, ZINM10, ZINM20, and ZINM30 are 1298.84 m^2^g^−1^, 108.85 m^2^g^−1^, 1282.26 m^2^g^−1^, 1191.31 m^2^g^−1^, and 1099.89 m^2^g^−1^, respectively. When compared to ZIF67, the ZINM30 sample shows a relatively lower surface area (1099.89 m^2^g^−1^); however, it has a much higher pore volume (0.160 cm^3^/g) than that of ZIF67 (0.036 cm^3^/g). This might be mainly attributed to the formation of a 3D-interconnected mesoporous framework in the heterojunction photocatalyst. The mesoporous characteristic could arise from the voids in the NiMoO_4_ microflowers, as verified by the FESEM images (Figure 3) [47]. The presence of microflowers could reduce the surface area and microporous nature of ZIF67. The above findings confirmed the successful formation of the ZINM heterojunction photocatalysts, among which ZINM30 contributes to abundant active sites to permit more light for the degradation of pollutants owing to its hierarchical micro- and mesoporous texture and large surface area.

### 3.5. Optical Absorption and Photoluminescence

**Figure 5:**
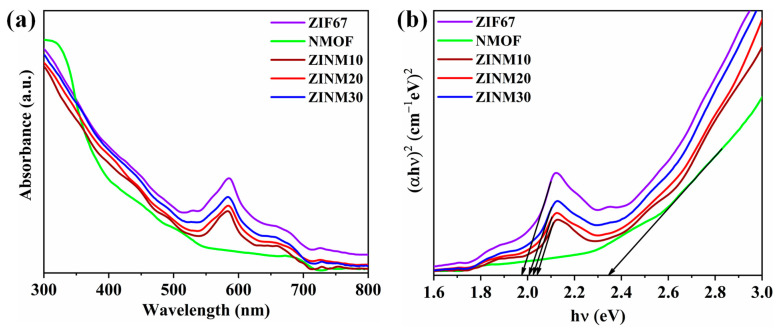
(**a**) UV-Vis absorbance. (**b**) (*αhν*)^2^ versus hν plots of ZIF67, NMOF, ZINM10, ZINM20, and ZINM30 photocatalysts.

The light absorption properties of ZIF67, NMOF, and ZINM30 photocatalysts were evaluated using UV-Vis spectroscopy. As depicted in Figure 5a, NMOF has an absorption edge at ~390 nm and is capable of harvesting light in the UV and visible regions. Meanwhile, ZIF67 has a broad absorption feature in the UV region (<400 nm) due to the ligand-to-metal charge transfer (LMCT) character [48]. Moreover, the material exhibits an absorption maximum at 585 nm with two shoulder peaks at 530 nm and 560 nm, which can be credited to the higher-lying [^4^A_2_(F) → ^4^T_1_(P)] d-d ligand field transitions of tetrahedrally coordinated Co^2+^ [49,50]. As expected, the loading of NMOF in ZIF67 favored the ZINM heterojunctions to possess the capability to absorb light in the UV and visible regions. The band gap energy (*E_gap_*) was computed using (*αhν)* = *B*(*hν* − *E*_gap_)*^n^*^/2^, where *ν*, *h*, *B,* and *α* represent the photon frequency, the Planck constant, a constant, and the absorption coefficient, respectively. Additionally, the factor ‘n’ relies on the electron transition characteristics and is set to 1 for direct transitions in ZIF67 and NiMoO_4_ [51,52,53]. The *E*_gap_ values of photocatalysts were determined using the plots of (*αhν*)^2/^*^n^* versus photon energy (*hν*), as presented in Figure 5b. Consequently, the estimated band gap energies of ZIF67, NMOF, ZINM10, ZINM20, and ZINM30 were about 1.98, 2.35, 2.04, 2.02, and 2.01 eV, respectively. The band gap was decreased due to the reduced crystallite size and the hierarchical morphology of both ZIF67 and NMOF in the composites. The results implied that ZINM samples can be reckoned as visible-light-driven photocatalysts.

Furthermore, PL spectra of the photocatalysts were collected to examine the recombination rate of photoexcited e^−^-h^+^ pairs, using an excitation wavelength of 370 nm. These charge carriers must be efficiently separated and transported to achieve high photocatalytic activity [54]. Typically, a higher PL intensity implies a higher recombination rate of the photoexcited charge carriers [55]. The room temperature PL emission spectra of ZIF67, NMOF, ZINM10, ZINM20, and ZINM30 photocatalysts are depicted in Figure S3. A strong PL emission peak around 455 nm is noticed for ZIF67, suggesting rapid recombination of photogenerated charge carriers. In contrast, NMOF displays an emission peak at 426 nm. Interestingly, ZINM30 shows a significantly lower emission intensity compared to the individual NMOF and ZIF67 components, likely due to the formation of a heterojunction between them. This effective combination promotes the separation of photoexcited e^−^-h^+^ pairs, reducing recombination and enhancing the photocatalytic performance of ZINM heterojunctions. Interestingly, the emission intensity of ZINM composites decreases with increasing NMOF content, indicating improved charge separation and longer charge carrier lifetimes. Based on these observations, it can be expected that the synthesized photocatalysts will significantly improve photocatalytic efficiency.

### 3.6. Photoelectrochemical Analysis

**Figure 6:**
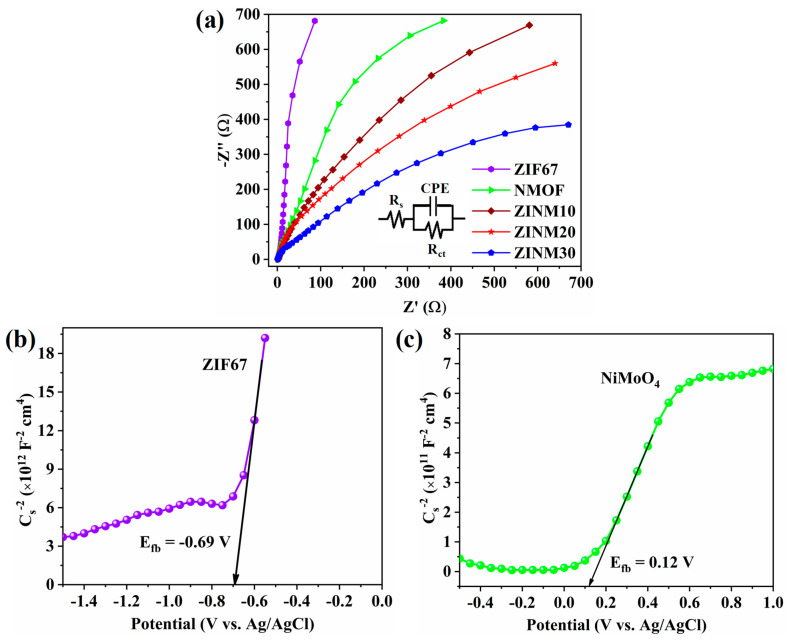
(**a**) EIS Nyquist plots of ZIF67, NMOF, ZINM10, ZINM20, and ZINM30 photocatalysts (the inset shows the equivalent electrical circuit). Mott–Schottky plots of (**b**) ZIF67 and (**c**) NMOF.

Electrochemical impedance spectrum (EIS) of the photocatalysts was performed to obtain more information about the interfacial charge transfer and separation efficiency of photoexcited charge carriers. In a typical Nyquist plot, the arc radius represents the interfacial layer resistance at the electrode surface, charge transfer of the material, and carrier recombination kinetics. A smaller arc radius reflects lower charge transfer resistance and a better separation rate of photoexcited charge carriers [56]. The fitting of EIS plots was performed as per the equivalent circuit shown in the inset of Figure 6a. The calculated values of series resistance (R_s_), charge transfer resistance (R_ct_), and constant phase element (*CPE*) are presented in Table S3. Figure 6a demonstrates that the ZINM30 photocatalyst exhibits the smallest arc radius, indicating its lowest charge transfer resistance (1.438 × 10^3^ Ω). The performance of ZINM30 photocatalyst improved because of the introduction of NMOF on the surface of ZIF67, which not only promotes the migration of photoexcited charge carriers but also hampers their rapid recombination, thereby increasing the effectiveness of photocatalytic degradation. These EIS outcomes are in line with the PL spectral results.

The performance of a semiconductor photocatalyst typically relies on its energy band structure. Hence, to identify the band position and type of semiconductor conductivity, the Mott–Schottky plot was employed. Figure 6b,c displays the corresponding plots of ZIF67 and NiMoO_4_. Since both ZIF67 and NiMoO_4_ show positive slopes, they are n-type semiconductors [57]. The flat-band potential (E_fb_) was determined by extending the linear part of 1/C_s_^2^ to the x-axis. The E_fb_ value was −0.69 V (versus Ag/AgCl) for ZIF67 and 0.12 V for NiMoO_4_. Since the CB edge (E_CB_), for n-type semiconductors, is located typically 0.1–0.3 V above the position of E_fb_, the E_CB_ values of ZIF67 and NiMoO_4_ were computed to be −0.59 V and 0.22 V (versus NHE), respectively [58,59]. From the E_gap_ of ZIF67 (1.98 eV) and NiMoO_4_ (2.35 eV), the VB edges (E_VB_) were estimated as 1.39 V and 2.57 V (versus NHE), respectively [59].

### 3.7. Photodegradation of Antibiotic Pollutants

**Figure 7:**
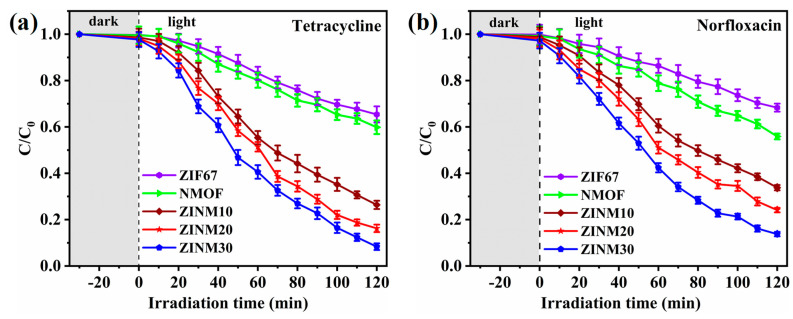
Photodegradation measurements of TCE and NF using the pure ZIF67, NMOF, and ZINM heterojunctions under visible light: C/C_0_ versus irradiation time plots for (**a**) TCE and (**b**) NF (the error bars represent the standard deviation over three repeated experiments).

To evaluate the performance of the as-synthesized photocatalysts, TCE and NF were used as targeted pollutants. In Figure 7, all ZINM heterojunction samples exhibited higher degradation rates than pure NMOF for both pollutants, indicating the significance of ZIF67 in improving the performance of NMOF. As can be seen in Figure 7a, the TCE degradation rates were about 34.59% and 40.14% for pure ZIF67 and NMOF samples, respectively. As the NMOF content increased, the photocatalytic performance of the ZINM heterojunctions increased as well. The ZINM30 possessed the optimal performance over TCE, and the degradation rate was about 91.67% after 120 min of light irradiation. This improvement can be credited to the heterojunction formed between ZIF67 and NMOF, which permits the fast transfer of photoexcited electrons, thereby improving the photogenerated charge carriers’ separation rate. It is necessary to explain the factors responsible for enhanced photocatalytic activity of ZINM heterojunctions. The light absorption and photocatalytic properties strongly depend on several factors, such as surface area, morphology, particle size, and crystallinity. As is verified from SEM images (Figure 3c–e), the rhombic dodecahedral ZIF67 shows relatively abundant micropores with NMOF anchored on its surface. The 3D flower-like NMOF is self-assembled by sheet-like structures, which is conducive to increasing the specific surface area of the photocatalyst and generating more active adsorption sites. The thin sheet-like structures in NiMoO_4_ microflowers favor the photogenerated carriers to reach the photocatalysts’ surface and participate in photocatalytic reactions. Moreover, due to its increased BET surface area (1099.89 m^2^/g), ZINM30 can provide abundant active sites during photocatalytic reactions, producing more photoexcited electron–hole pairs. With a highly porous feature (14.11 nm, 0.160 cm^3^/g), ZINM30 can capture more TCE/NF molecules onto their surfaces, enhancing photocatalytic reactivity. Benefitting from the sheet-like structures in NMOF, the porous structure can suppress the recombination of electrons and holes during the charge migration. Thus, the high surface area and porosity synergistically contribute to the boosted photocatalytic activity of the ZINM30 photocatalyst. The optimal loading of NMOF onto ZIF67 is beneficial to boost the photocatalyst’s specific surface area and active sites, allowing for more absorption of TCE. Similarly, the heterojunction samples also showed high degradation efficiency for NF. When the loading of NMOF increased from 10 wt.% to 30 wt.%, the final degradation efficiency reached a maximum of 86.23% for ZINM30, which was higher than the values of pure ZIF67 (31.67%), NMOF (44.11%), or other composite counterparts (Figure 7b). The photocatalytic performance of the as-synthesized Z-scheme heterojunction was compared with some previously reported works, which are presented in Table S4 [60,61,62,63,64,65].

**Figure 8:**
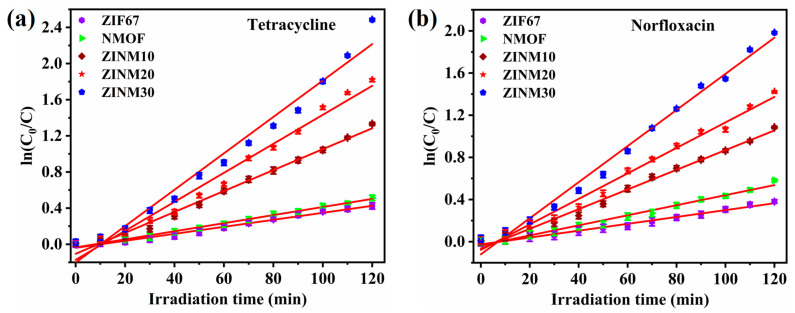
First-order kinetics plot for the photodegradation of (**a**) TCE and (**b**) NF (the error bars represent the standard deviation of three repetitions).

**Figure 9:**
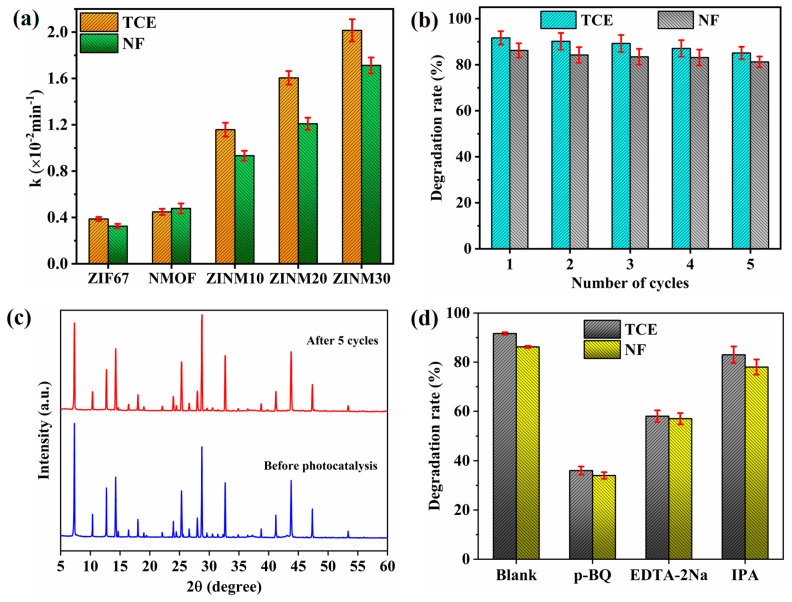
(**a**) The rate constants of different photocatalysts in the degradation of TCE and NF. (**b**) Reusability of ZINM30 for the TCE and NF degradation. (**c**) XRD pattern of ZINM30 before and after the photocatalytic reaction. (**d**) Photodegradation of antibiotic pollutants by ZINM30 photocatalyst in the presence of various scavengers (the error bars represent the standard deviation of three repetitions).

The reaction kinetics of the photocatalysts during the degradation of TCE and NF were explored. It is obvious from Figure 8a,b that the degradation process of pollutants obeys the pseudo-first-order kinetics model. Figure 9 shows the rate constants, reusability, stability, and scavenger tests of the photocatalysts. The rate constants (k) extracted from curve fitting are shown in Figure 9a. The optimal rate constants for the elimination of TCE and NF by ZINM30 were estimated as 0.02015 min^−1^ and 0.01713 min^−1^, respectively. These k values were ~5.2-fold greater than the values of the NMOF sample (0.00387 min^−1^ and 0.00325 min^−1^). This indicates a higher reaction rate and superior catalytic activity of the composite samples in degrading the pollutants.

Recycling tests were conducted on the ZINM30 composite to assess its reusability and stability (Figure 9b). After five consecutive cycles, the composite’s performance slightly declined and the degradation rates persisted at about 85.1% (TCE) and 81.2% (NF). This suggests that the ZINM30 composite holds good stability for antibiotic degradation. The performance reduction may be due to the loss of some active chemical groups and the residual intermediates generated during the pollutant degradation, which could partially cover the active sites of the heterojunction composite. Furthermore, the stability of ZINM30 was characterized using XRD. As seen in Figure 9c, the diffraction peaks of the ZINM30 composite in the XRD spectra were slightly weakened after five cycles, but the peak positions remained mostly unchanged. This is good evidence for the excellent durability and reusability of the composite.

To further study the degradation mechanism followed by the photocatalysts, the highly reactive photogenerated species involved in the degradation process were assessed using various scavengers. The role of reactive species such as hydroxyl radical (•OH), superoxide radical (•O_2_^−^), and photogenerated hole (h^+^) in the photocatalytic process of the antibiotic pollutants (TCE and NF) was analyzed by employing IPA, *p*-BQ, and EDTA-2Na as free radical capture agents, respectively. The influence of these scavengers on the pollutant removal efficiency of ZINM30 heterojunction photocatalyst under visible light irradiation is compared in Figure 9d. All three reactive species participated in the degradation of TCE and NF. The degradation efficiency of ZINM30 was reduced to 36% and 58%, respectively, for *p*-BQ and EDTA-2Na, as their addition impeded the photodegradation of TCE. Similarly, the degradation efficiencies of ZINM30 in removing NF were about 34% (for *p*-BQ) and 57% (for EDTA-2Na), respectively. This implies that the inhibition effect of *p*-BQ is higher than that of EDTA-2Na. After adding *p*-BQ, the degradation of pollutants was greatly inhibited because *p*-BQ may remove O_2_ dissolved in the solution and restrict the generation of •O_2_^−^. Nonetheless, IPA had a minimal effect on the photocatalytic activity of the ZINM30 sample, with degradation efficiencies of about 83% (TCE) and 78% (NF). This indicates that the •OH radical would not be seriously involved in the photocatalytic activity because the VB potentials of ZIF67 and NMOF are far lower than those required for the •OH formation, as evident from the Mott–Schottky plot. Consequently, the influence of reactive species can be ordered as •O_2_^−^ > h^+^ > •OH. These findings demonstrate that •O_2_^−^ and h^+^ are key components of the photocatalytic process, while the removal efficiency is relatively unaffected by •OH.

### 3.8. Description of Degradation Mechanism

**Figure 10:**
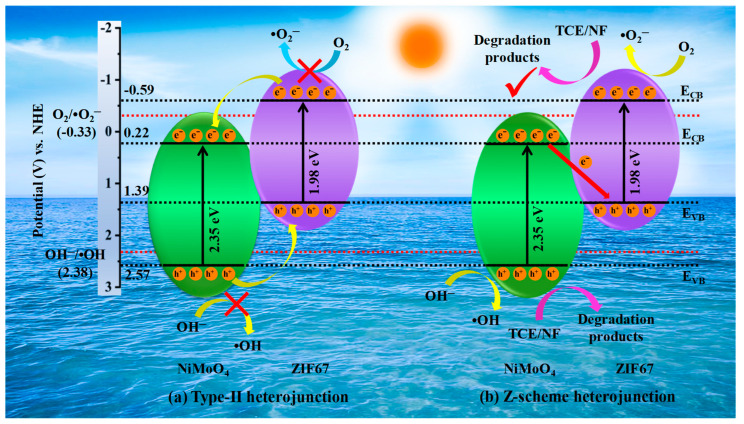
Illustration of charge transfer mechanisms in (**a**) type-II heterojunction and (**b**) Z-scheme ZIF67/NiMoO_4_ heterojunction photocatalysts.

Two probable mechanisms of charge transfer, viz., type-II and Z-scheme, are proposed based on experimental findings to assess how the antibiotics degrade in water. As illustrated in Figure 10a, assuming the traditional type-Ⅱ heterojunction mechanism, under light irradiation, the photoexcited electrons are transported from the CB of ZIF67 (SC-1) to the CB of NiMoO_4_ (SC-2), whereas the photoexcited holes are migrated from the VB of NiMoO_4_ to the VB of ZIF67. In this context, the CB electrons in NiMoO_4_ are inadequate to create •O_2_^−^, as the E_CB_ of NiMoO_4_ (0.22 eV) is relatively larger (more positive) than the reduction potential of O_2_/•O_2_^−^ (−0.33 eV). Meanwhile, the E_VB_ of ZIF67 (1.39 eV) is relatively lower (less positive) than the oxidation potential of OH^−^/•OH (2.38 eV). The aforementioned type-Ⅱ carrier transport does not comply with the radical capture test results. Another possible strategy is the Z-scheme mechanism (Figure 10b), through which the degradation mechanism can be explained. According to the Z-scheme, when the composite photocatalyst is exposed to light, electrons are excited from the VB to the CB, leaving the VB with an equal number of holes behind. This results in the generation of photoexcited electron–hole pairs (e^−^-h^+^). The as-accumulated photoexcited electrons in the lower CB position of NiMoO_4_ can recombine with holes in the higher VB position of ZIF67 at the heterojunction interface [66]. Given that the E_CB_ of ZIF67 is −0.59 eV, which is less (more negative) than the reduction potential of O_2_/•O_2_^−^ (−0.33 eV), the CB electrons of ZIF67 can be easily captured by the absorbed O_2_ molecules, generating •O_2_^−^ radicals. Likewise, OH^−^ anions are oxidized to produce •OH species since the E_VB_ of NiMoO_4_ (2.57 eV) is larger (more positive) than the oxidation potential of OH^−^/•OH (2.38 eV). However, because holes in the VB of NiMoO_4_ possess strong oxidative capability, they can directly oxidize and degrade antibiotic compounds. Thus, the photocatalytic degradation process is contributed by three reactive species, h^+^, •O_2_^−^, and •OH, aligning with the results of the trapping test. Consequently, the ZIF67/NiMoO_4_ heterojunction composite followed the Z-scheme mechanism to degrade antibiotics (TCE or NF) with enhanced photocatalytic activity. This mechanism promotes the separation efficacy of photoexcited charge carriers and keeps highly active oxidative and reductive sites, leading to the creation of more active species.

## 4. Conclusions

NiMoO_4_ microflower-loaded ZIF67 composites were rationally prepared via a facile solution–coprecipitation approach. The as-synthesized Z-scheme heterojunction photocatalyst was applied to degrade TCE and NF antibiotics. Among the individual and composite photocatalysts, ZINM30 exhibited superior photocatalytic efficiency; the degradation rate reached 91.67% for TCE and 86.23% for NF in 120 min under visible light. The kinetic rate constants of ZINM30 were ~ 5.2 times greater than those of the NMOF sample. This performance boost is attributed to its reduced band gap (E_gap_ = 2.01 eV), superior BET surface area (1099.89 m^2^/g), efficient charge carrier separation, and lower charge transfer resistance (1.438 × 10^3^ Ω). Moreover, the ZINM30 composite was highly stable for up to five cycles. The radical trapping test revealed that *h*^+^ and •O_2_^−^ were the primary species; meanwhile, •OH also participated in the degradation of antibiotics. The intimate contact and matching of the energy bands between ZIF67 and NiMoO_4_ are the main causes for the improved photocatalytic performance. The proposed Z-scheme heterojunction photocatalyst is a potential candidate for the effective degradation of antibiotics in the context of environmental purification.

## Data Availability

Data are contained within the article and Supplementary Materials. Further inquiries can be directed to the corresponding author.

## Supplementary Materials

The following supporting information can be downloaded at https://www.mdpi.com/article/10.3390/ma17246225/s1: Characterization; Photoelectrochemical measurements; Photocatalytic activity test; Calculation of structural parameters; Figure S1: Schematic for the synthesis of the photocatalysts; Figure S2: W-H plots for the photocatalysts; Figure S3: High-resolution spectra of (a) C1s and (b) N 1s of ZINM30 photocatalyst; Figure S4: Particle size distribution histogram for ZIF67. Figure S5: Photoluminescence spectra of the photocatalysts; Table S1: Lattice constants, crystallite size, and microstrain of the photocatalysts; Table S2: BET surface area, pore width, and pore volume of the photocatalysts; Table S3: EIS fitting parameters of the photocatalysts; Table S4: A comparison of various Z-scheme heterojunctions for photocatalytic degradation of tetracycline and norfloxacin.
