# Supplementary material for: Construction of Z-Scheme ZIF67/NiMoO4 Heterojunction for Enhanced Photocatalytic Degradation of Antibiotic Pollutants
Source: Materials (Basel). 2024 Dec 20;17(24):6225. doi: 10.3390/ma17246225 (PMC11676704; doi:10.3390/ma17246225)
Supplement: Supplementary file 1 [file materials-17-06225-s001.zip › materials-3359068-supplementary.pdf]

Supporting information

Characterization

The crystal structure of photocatalysts was investigated using an X-ray diffractometer (XRD, Rigaku) supplied with a CuK_{α} (λ = 0.15406 nm) source operated in a 2θ = 5°–60° scan range in steps of 0.02°/min. Functional groups were identified using a Fourier transform infrared spectrometer (FTIR-4600, JASCO) in the 400–4000 cm^{-1} spectral range. The microstructure and energy dispersive X-ray (EDX) studies were carried out with a field-emission scanning electron microscope (FESEM, Hitachi S-4800) at 30 kV accelerating voltage. Chemical compositions and valence states were analyzed using an X-ray photoelectron spectroscopy (XPS, Multilab-2000, Thermo Scientific) apparatus, equipped with a monochromatic AlK_{α} X-ray source (1486.6 eV) for excitation. N_2 adsorption-desorption isotherms were acquired using a gas sorption analyzer at 77 K (ASAP 3020, Micromeritics). The specific surface areas were evaluated using the multi-point BET (Brunauer-Emmett-Teller) method. The pore volumes and size distributions were estimated using the Barrett-Joyner-Halenda (BJH) model. Optical absorption spectra were achieved by a UV-Vis spectrophotometer (V-770, JASCO). Photoluminescence (PL) spectra were produced by a spectrofluorimeter (FluoroMate FS-2, SCINCO) under 370 nm excitation.

Photoelectrochemical measurements

Electrochemical impedance spectrum (EIS) and Mott-Schottky analysis were performed on an electrochemical workstation (CHI760E) equipped with a typical three-electrode setup under the Xenon lamp (300 W, $\lambda > 420$ nm) irradiation. Each photocatalyst (10 mg) was dispersed in 1 mL of EtOH and then sonicated for 10 min. The as-produced slurry was spin-coated on an ITO glass surface (indium tin oxide, 2 × 1 cm^2 area) and dried overnight at 60 °C. The photocatalyst-loaded ITO plate (1 × 1 cm^2 active area), Pt wire, and Ag/AgCl served as working-, counter-, and reference-electrodes, respectively. The electrolyte used was

aqueous Na_2SO_4 (0.5 M, 30 mL). Mott-Schottky measurements were done at a frequency of 1 kHz. Impedance data was obtained in the 10^5–0.1 Hz frequency range, with a 5 mV perturbation at the open circuit potential. The Mott-Schottky plot can describe the space-charge capacitance (C_s) that varies across the depletion region for the applied potential (E_a) as,

$$\frac{1}{C_s^2} = \frac{2}{e\varepsilon\varepsilon_0 A^2 N_d}\left(E_a - E_{fb} - \frac{k_B T}{e}\right) \quad \text{(S1)}$$

where e, ε, ε_0, A, N_d, E_a, E_{fb}, T, and k_B denote the electronic charge, dielectric constant, permittivity in free space, active area of electrode, concentration of charge carriers, applied potential, flat-band potential, temperature (in Kelvin), and the Boltzmann's constant, respectively.

Photocatalytic activity test

Photocatalytic degradation tests of TCE and NF were accomplished in a photoreactor with a 300 W xenon lamp as a visible light source. The initial concentration of photocatalysts was 0.5 mg/mL in water, and 5×10^{-5} M of TCE (or NF) was mixed with it. Before the reaction, the solution was sonicated for 5 min to improve dispersion. Then, it was placed in the photoreactor and magnetically stirred. To assess the effect of adsorption on pollutant removal, the solutions were initially placed in a dark environment for about 30 min. During the light irradiation process, 5 mL of solution was extracted at equal time intervals, and centrifuged at 8000 rpm for 10 min. The supernatant was filtered through a 0.22 μm polytetrafluoroethylene (PTFE) membrane filter, and the pollutants in the filtrate were qualitatively analyzed by measuring the absorbance of the filtrate using a UV-Vis spectrometer. Similarly, the absorbance values of all TCE (or NF) samples subjected to irradiation for up to 120 min were recorded. The photodegradation efficiency was evaluated by,

$$\%\ \text{degradation} = \left(\frac{C_0 - C}{C_0}\right) \times 100 \quad \text{(S2)}$$

If there is only a variation of pollutant concentration vs. time, the rate of photocatalytic degradation can be expressed by the pseudo-first-order reaction kinetics, which is represented as

$$ln\left(\frac{C_0}{C}\right) = kt \qquad \text{(S3)}$$

where C_0 denotes the pollutant's concentration at t = 0, C represents the pollutant's concentration at any time t, and k means the rate constant. In addition to that, the role of •OH, $•O_2^-$, and h^+ radicals in the photodegradation process was assessed using isopropanol (IPA), *p*-Benzoquinone (*p*-BQ), and ethylenediaminetetraacetic acid disodium salt (EDTA-2Na), respectively, as free radical scavengers. All the photocatalytic tests were repeated three times (n = 3) under analogous conditions.

Calculation of structural parameters

The lattice constants (a, b, and c) were calculated by

$$\frac{1}{d^2} = \frac{1}{sin^2\beta}\left(\frac{h^2}{a^2} + \frac{k^2 sin^2\beta}{b^2} + \frac{l^2}{c^2} - \frac{2hlcos\beta}{ac}\right) \qquad \text{(S4)}$$

The average crystallite size of the samples was calculated using the Debye-Scherrer equation, which is represented by

$$D = \frac{k\lambda}{\beta cos\theta} \qquad \text{(S5)}$$

where D is the crystallite size, k = 0.89, λ is the X-ray wavelength (0.15406 nm), β is the full width at half maximum (FWHM), and θ is the diffraction angle. The average microstrain (ε) was determined using,

$$\varepsilon = \frac{\beta cos\theta}{4} \qquad \text{(S6)}$$

For comparison, the average values of D and ε were also estimated using the Williamson-Hall equation,

$$\beta cos\theta = \frac{k\lambda}{D} + 4\varepsilon sin\theta \qquad \text{(S7)}$$

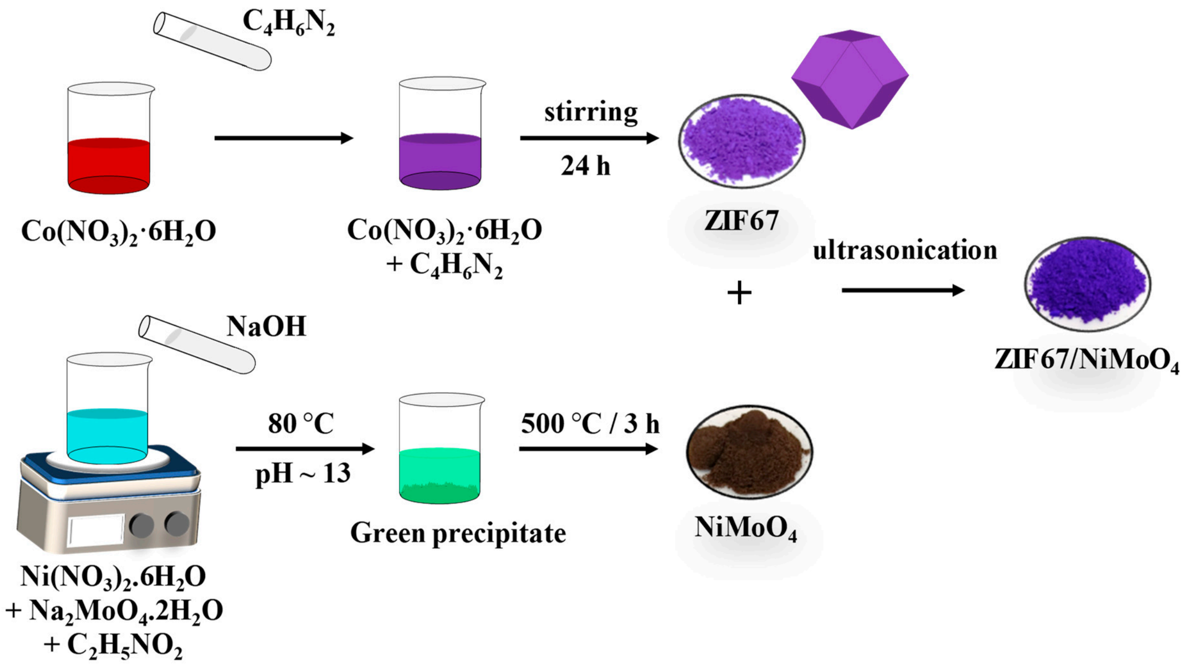

Figure S1. Schematic for the synthesis of the photocatalysts.

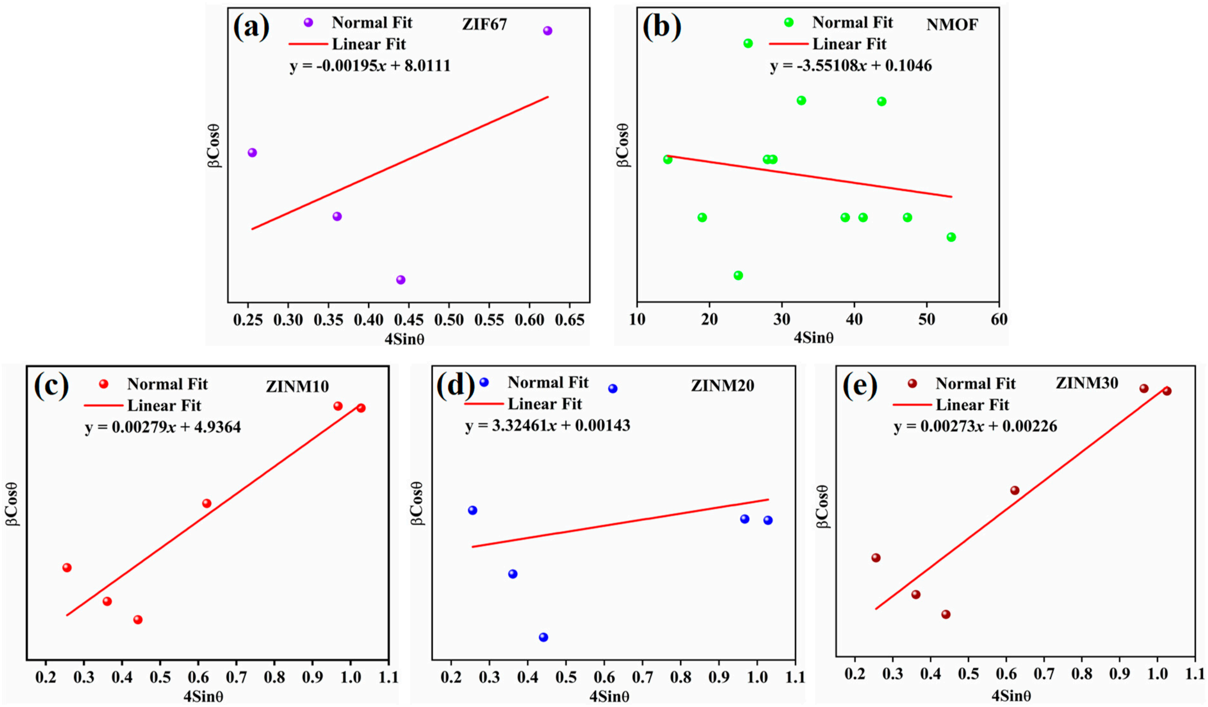

Figure S2. W-H plots for the photocatalysts.

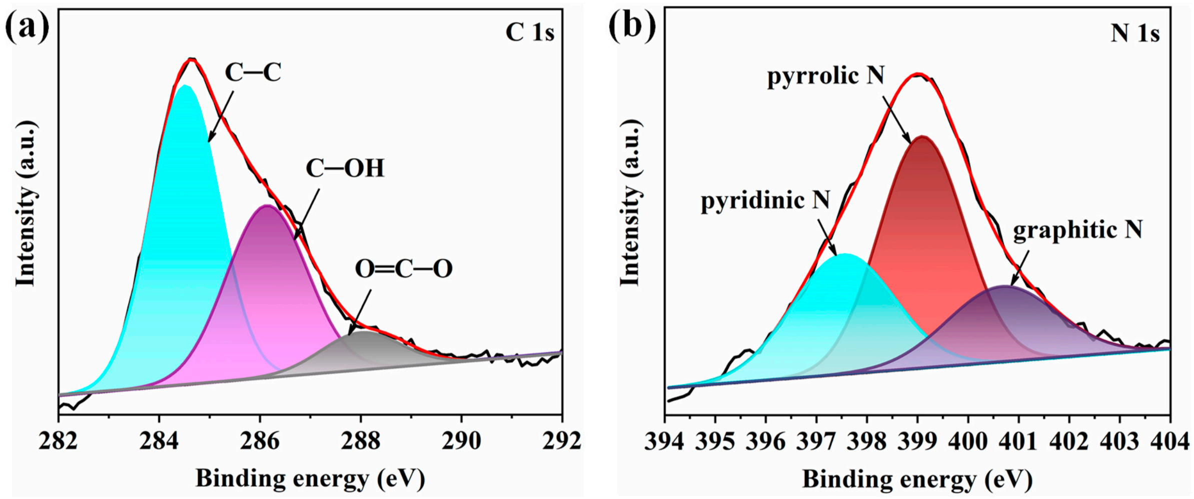

Figure S3. High-resolution spectra of (a) C1s and (b) N 1s of ZINM30 photocatalyst.

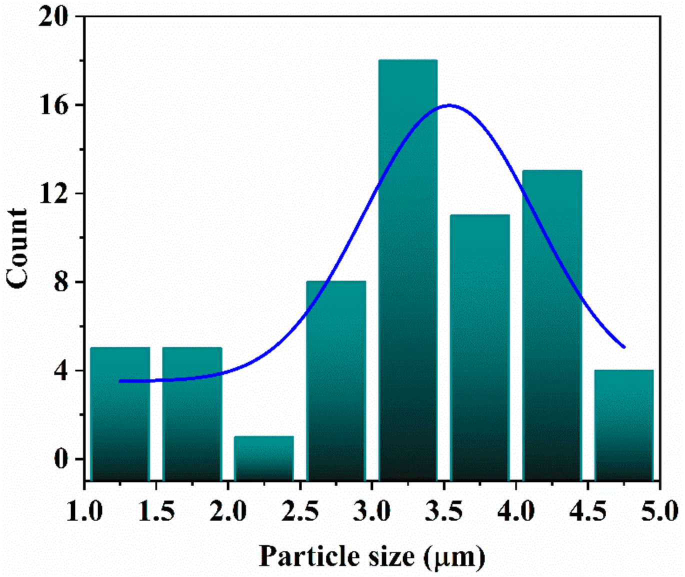

Figure S4. Particle size distribution histogram for ZIF67.

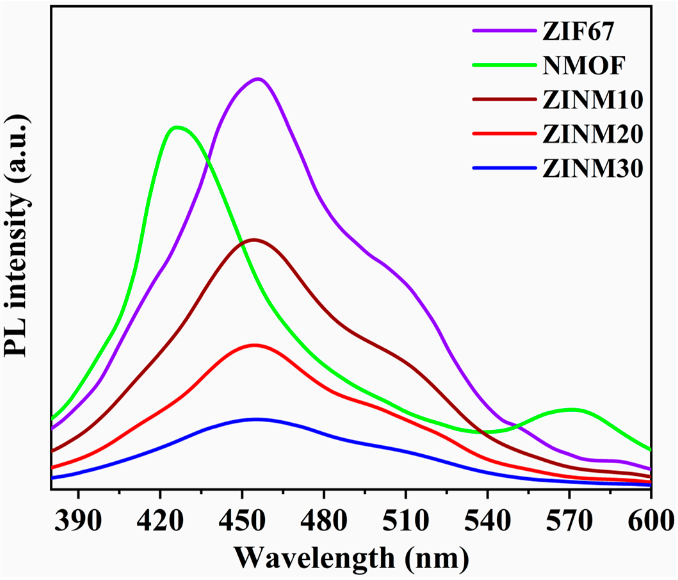

Figure S5. Photoluminescence spectra of the photocatalysts.

Table S1. Lattice constants, average crystallite size, and average microstrain of the photocatalysts.

Photocatalyst	Lattice constants			Average crystallite Size (D) (nm)		Average microstrain (ε)	
	a	b	c	Debye-Scherrer	W-H plot	Debye-Scherrer	W-H plot
ZIF67	9.421	8.728	7.661	39.28	18.08	0.162	0.195
NMOF	8.213	8.223	6.124	18.85	13.84	0.465	0.355
ZINM10	9.143	8.010	7.221	56.23	14.28	0.245	0.276
ZINM20	9.352	7.002	7.544	46.60	12.90	0.330	0.280
ZINM30	9.684	8.254	7.220	23.22	11.07	0.588	0.324

Table S2. BET surface area, pore width, and pore volume of the photocatalysts.

Photocatalyst	Surface area S_{BET} (m^2/g)	Average pore size D_{Avg} (nm)	Pore volume V_{Total} (cm^3/g)
ZIF67	1298.84	8.51	0.036
NMOF	108.85	20.01	0.648
ZINM10	1282.26	9.97	0.060
ZINM20	1191.31	12.25	0.126
ZINM30	1099.89	14.11	0.160

Table S3. EIS fitting parameters of the photocatalysts.

Photocatalyst	R_s (Ω)	CPE (mF/cm^2)	R_{ct} (Ω)
ZIF67	3.096	0.851	1.140×10^4
NMOF	2.287	0.782	9.437×10^3
ZINM10	2.753	0.699	5.652×10^3
ZINM20	2.141	0.647	3.703×10^3
ZINM30	1.695	0.594	1.438×10^3

Table S4. A comparison of various Z-scheme heterojunctions for photocatalytic degradation of tetracycline and norfloxacin.

S. No.	Photocatalyst	Synthesis method	Morphology	Light source	Pollutant	Efficiency/ Duration	Ref.
1	$ZnO/ZnCo_2O_4$-Co_3O_4|Co	Solid-state chemical reaction	---	300 W Xe lamp	Norfloxacin	80.70% (180 min)	[55]
2	ZnO/g-C_3N_4	Hydrothermal	Nanorods/ nanosheets	300 W Xe lamp	Norfloxacin	86.7% (480 min)	[56]
3	AgCl/BiOCl	Water-bath method	Nanoparticles/ nanosheets	350 W Xe lamp	Norfloxacin	74.37% (60 min)	[57]
4	Fe/Mn-MOF@CdS	Solvothermal/ hydrothermal	Square-conical	300 W Xe lamp	Tetracycline	90.95% (160 min)	[58]
5	Fe_3O_4/BiOBr/BiOI	Solvothermal	Nanoparticles/ microspheres	300 W Xe lamp	Tetracycline	87% (80 min)	[59]
6	SnS_2/Bi_2MoO_{6-x}	Hydrothermal	Nanoparticles/ hollow microspheres	300 W Xe lamp	Tetracycline	89% (90 min)	[60]
7	ZIF67/$NiMoO_4$	Solvothermal	Microflowers	300 W Xe lamp	Tetracycline and Norfloxacin	91.67% and 86.23% (120 min)	**This work**
